# A mega-analysis of expression quantitative trait loci in retinal tissue

**DOI:** 10.1371/journal.pgen.1008934

**Published:** 2020-09-01

**Authors:** Tobias Strunz, Christina Kiel, Felix Grassmann, Rinki Ratnapriya, Madeline Kwicklis, Marcus Karlstetter, Sascha Fauser, Nicole Arend, Anand Swaroop, Thomas Langmann, Armin Wolf, Bernhard H. F. Weber

**Affiliations:** 1 Institute of Human Genetics, University of Regensburg, Regensburg, Germany; 2 Institute of Medical Sciences, University of Aberdeen, Aberdeen, United Kingdom; 3 Neurobiology-Neurodegeneration & Repair Laboratory, National Eye Institute, Bethesda, United States of America; 4 Laboratory for Experimental Immunology of the Eye, Department of Ophthalmology, Faculty of Medicine and University Hospital Cologne, Cologne, Germany; 5 Roche Innovation Center Basel, F. Hoffmann-La Roche Ltd, Basel, Switzerland; 6 Department of Ophthalmology, Ludwig-Maximilians-University, Munich, Germany; 7 Department of Ophthalmology, University of Ulm, Ulm, Germany; 8 Institute of Clinical Human Genetics, University Hospital Regensburg, Regensburg, Germany; Augusta University, UNITED STATES

## Abstract

Significant association signals from genome-wide association studies (GWAS) point to genomic regions of interest. However, for most loci the causative genetic variant remains undefined. Determining expression quantitative trait loci (eQTL) in a disease relevant tissue is an excellent approach to zoom in on disease- or trait-associated association signals and hitherto on relevant disease mechanisms. To this end, we explored regulation of gene expression in healthy retina (n = 311) and generated the largest cis-eQTL data set available to date. Genotype- and RNA-Seq data underwent rigorous quality control protocols before FastQTL was applied to assess the influence of genetic markers on local (cis) gene expression. Our analysis identified 403,151 significant eQTL variants (eVariants) that regulate 3,007 genes (eGenes) (Q-Value < 0.05). A conditional analysis revealed 744 independent secondary eQTL signals for 598 of the 3,007 eGenes. Interestingly, 99,165 (24.71%) of all unique eVariants regulate the expression of more than one eGene. Filtering the dataset for eVariants regulating three or more eGenes revealed 96 potential regulatory clusters. Of these, 31 harbour 130 genes which are partially regulated by the same genetic signal. To correlate eQTL and association signals, GWAS data from twelve complex eye diseases or traits were included and resulted in identification of 80 eGenes with potential association. Remarkably, expression of 10 genes is regulated by eVariants associated with multiple eye diseases or traits. In conclusion, we generated a unique catalogue of gene expression regulation in healthy retinal tissue and applied this resource to identify potentially pleiotropic effects in highly prevalent human eye diseases. Our study provides an excellent basis to further explore mechanisms of various retinal disease etiologies.

## Author summary

The retina is a multilayered and highly specified neural tissue crucial for high-resolution visual perception and spatial orientation. Environmental and genetic insults to the retina result in many blinding diseases, such as age-related macular degeneration or glaucoma. Commonly, many of these diseases are age-related suggesting that minor changes are accumulating over a life-time, with little or no contribution of strong individual effects. Specifically, this is true for genetic factors known to underlie the etiology of complex diseases including the prevalent eye diseases. In our study, we searched for effects on gene expression due to genetic variation using 311 healthy post-mortem retinal tissue samples. We show that 3,007 of the 16,766 genes investigated are regulated in the retina by genetic variations. Of these, 80 genes are potentially associated to one or more of twelve complex eye diseases or retinal traits tested. Interestingly, 10 genes appear to be involved in the development of several eye traits suggesting that cellular mechanisms may act at a common point in the disease process. Consequently, our study provides the basis to further explore retinal disease pathways and is likely to highlight target molecules for future therapeutic applications.

## Introduction

Genome-wide association studies (GWAS) reveal a contribution of common genetic variation to the etiologies of an ever increasing number of adult-onset diseases [1]. Despite such advances, mapping association signals to defined genomic regions most often fails to reveal the truly causative genetic variation and genes imperative to unravel underlying molecular disease mechanisms [2]. Bioinformatic follow up-studies are required to refine GWAS data to a curated set of genetic variants eventually pointing to genes to be defined as candidates for further analyses [3].

In this context, an attractive methodology is to link genetic variation to intermediate phenotypes, which may contribute to the disease etiology of interest. Such an approach takes advantage of identifying expression quantitative trait loci (eQTL), which offer an explanation for at least part of variation in mRNA expression in a tissue-specific manner [4]. Additionally, a plethora of GWAS data from a wide range of non-pathogenic phenotypes is available [1,5] allowing the correlation of eQTL variants (so-called eVariants) and their regulated genes (so-called eGenes) to intermediate phenotypes and thus potential phenotype altering mechanisms [6]. It should be noted that eQTL studies are usually performed in healthy tissue, although gene expression may change with disease onset or progression. Thus, conclusions from such studies about initial disease mechanisms need to be considered with caution. So far, only a single study investigated eQTL in retinal tissue with the majority of samples (312/406) retrieved from age-related macular degeneration (AMD) patients [7].

Here, we present a study combining eQTL data from three independent study sites to robustly characterize gene expression regulation in healthy retinal tissue. A subsequent analysis of eVariants that regulate several genes provides additional information on potentially shared genetic signals and thus shared disease mechanisms in the etiology of prevalent complex eye conditions [8,9]. Our data suggest the existence of such common mechanisms, thereby providing the basis to further explore disease pathways as well as future therapeutic applications.

## Results

### Mega-analysis of local eQTL

This study combines data from three sources and allows to explore the regulatory architecture of gene expression in healthy retinal tissue. After quality control (QC) (see Methods), 311 samples were included in the further analysis (**Table 1**).

**Table 1:** Study and sample summary.

Dataset	Human Genetics Regensburg	University Hospital Cologne	NEI Bethesda [7]
**Sample size before QC/ after QC**	161 / 143	78 / 74	105 / 94
**Mean Age**	59.0 (SD: 15.3)	69.2 (SD: 12.6)	74.2 (SD: 9.4)
**Gender (M / F)**	97 / 46	35 / 39	46 / 48
**RNA-Seq library**	NEXTFLEX Rapid Directional RNA-Seq Library Prep Kit	TruSeq Stranded mRNA Library Prep Kit	TruSeq Stranded mRNA Library Prep Kit
**RNA-Seq Sequencing platform**	Illumina HiSeq platform		
**RNA-Seq Sequencing depth**	20 m SE	50–80 m PE	10–20 m PE
**Read length**	83 bp	51 bp	125 / 126 bp
**Expressed genes (CPM > 1 in 10% of samples)**	18,290	18,971	18,401
**Expressed genes overlapping (autosomal)**	17,405 (16,766)		
**Genotyping Platform**	Custom HumanCoreExome BeadChip	Infinium OmniExpress-24 v1.2 BeadChip	UM_HUNT_Biobank v1.0 chip
**Imputed variants after QC**	8,686,883		
**eQTL (Q-Value < 0.05)**	580,170		
**eVariants (Q-Value < 0.05, unique)**	403,151		
**eVariants regulating several Genes (Q-Value < 0.05)**	99,165		
**eGenes (Q-Value <0.05, unique)**	3,007		
**Independent signals**	3,751		

CPM = Counts per million; QC = Quality control; SD = Standard deviation; SE = Single-end; PE = Paired-end

RNA sequencing (RNA-Seq) reads were initially analyzed separately per individual dataset. Expression threshold was set to counts per million (CPM) > 1 in at least 10% of samples per dataset. A total of 2,412 genes were exclusively found to be expressed in only one or two of the three datasets and subsequently excluded from further analysis. This should greatly enhance the stringency of true tissue expression and as such the detection of valid effects. Information about 17,405 genes shared between the datasets was then combined and normalized as detailed in the Methods. Of the combined dataset 16,766 genes were located on autosomes. Genotypes of the 311 retinal samples were imputed to the 1000 Genomes Project Phase 3 reference panel providing information on 8,686,883 quality-controlled variants (**Table 1**).

The merged genotype- and gene expression data were explored for local eQTL with a maximum variant-gene distance of 1 Mbp up-and downstream of the respective gene locus. After adjustment for multiple testing, we identified 580,170 significant eQTL (Q-Value < 0.05), which regulate 3,007 unique eGenes (**Table 1**). Altogether, 99,165 eVariants appear to regulate more than one eGene, resulting in 403,151 unique eVariants. The eQTL rs145885457—TSPAN11 showed the most significant nominal P-value (5.60 x 10^−123^), whereas rs141866579—*AL590399*.*1* revealed the highest but still significant association (P-value 2.51 x 10^−03^) in our analysis (**S1 Fig**).

The most significant eVariant for each eGene was used to perform an adjusted conditional analysis. With this approach, we detected additional 744 independent signals. The most significant eVariants of the primary analysis and the results of the conditional analysis were then compiled into a list of 3,751 independent signals (3,007 primary + 744 secondary) (**S1 Data**). Of note, the highly regulated genes *AGAP7P*, *CCK*, *KIF25*, *PTGR1*, *ADAMTS18*, and *AC108516*.*1* were each controlled by five independent signals.

### Characterization of gene expression regulation

**Fig 1:**
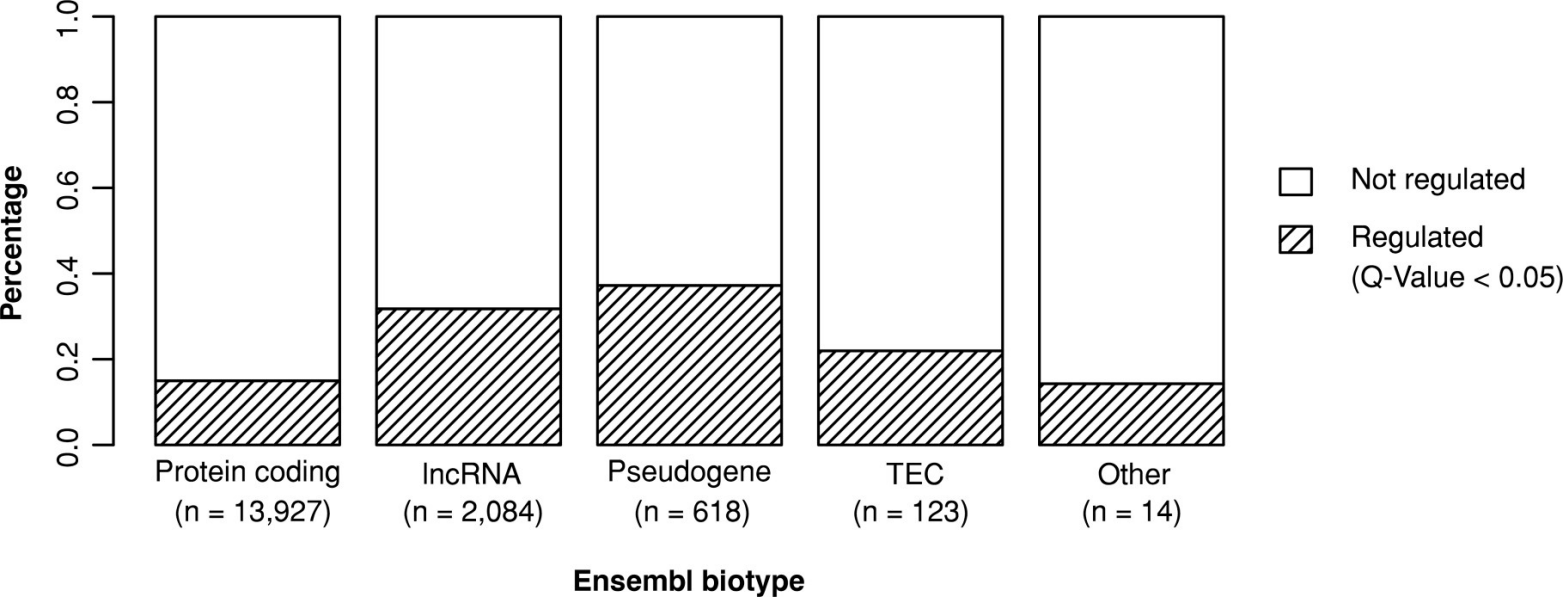
Distribution of Ensembl biotypes across all analyzed genes. 16,766 expressed autosomal genes in retinal tissue were characterized with regard to their Ensembl biotype [16]. The Q-Value threshold 0.05 was chosen to define significance for the eQTL analysis. For this graph, all biotypes including the term “Pseudogene” were compiled into one section. lncRNA = long non-coding RNA; TEC = to be experimentally confirmed.

A total of 3,007 (17.9%) of the 16,766 expressed autosomal genes were regulated by at least one eQTL. Remarkably, the percentage of regulated genes varied between Ensembl biotypes (**Fig 1**) from 14.98% (2,086 of 13,927, protein coding) to 37.21% (230 of 618, pseudogene). Furthermore, 31.77% of all long non-coding RNAs (662 of 2,084, lncRNAs) were significant eGenes.

**Fig 2:**
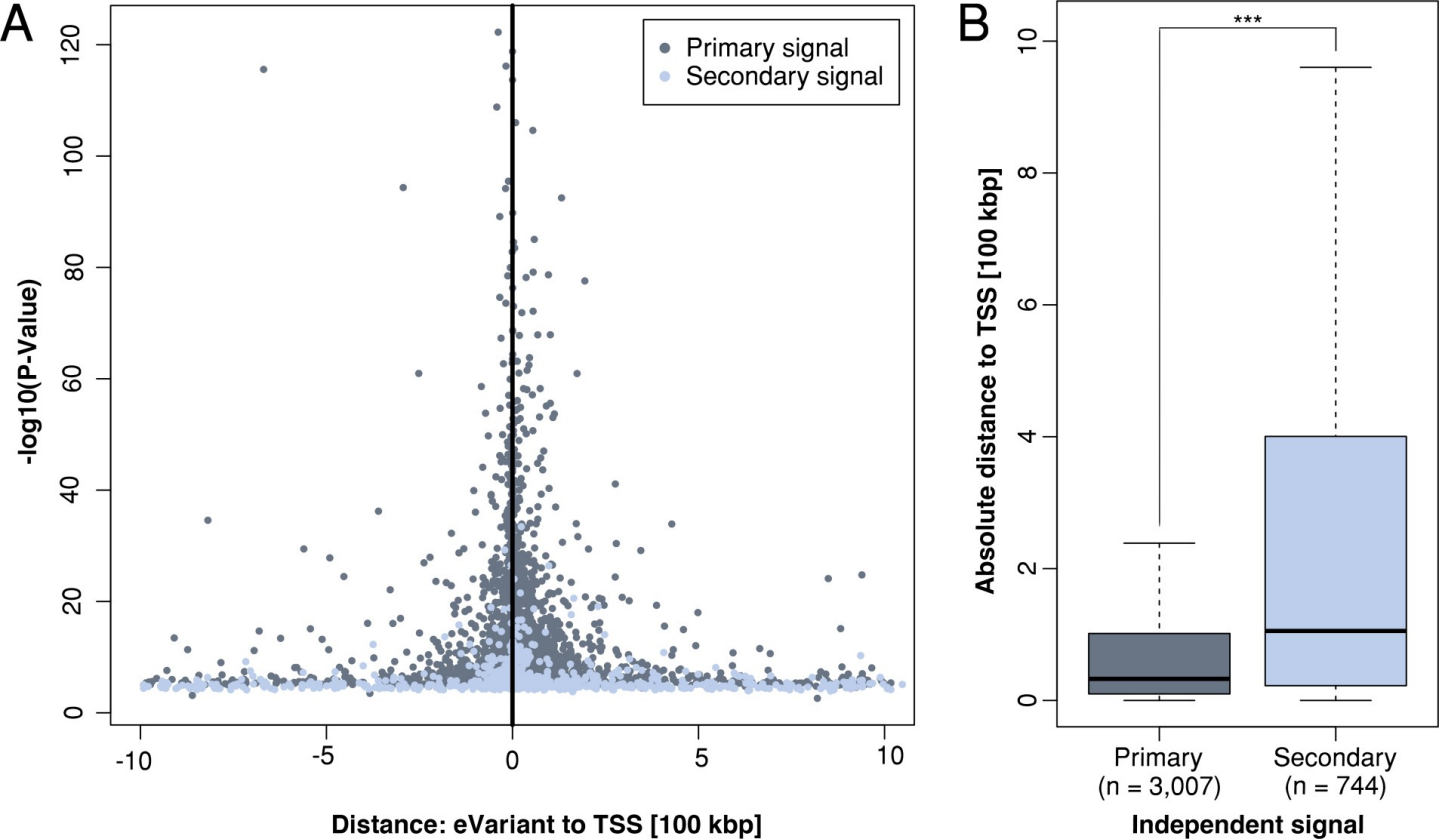
Genomic localization of eVariants. (**A**) The distance of the most significant eVariant to the transcription start site (TSS) of the respective eGene is plotted against the significance of the association (−log10 P-Value). Shown are the results of the primary analysis (primary signal, dark grey) and the most significant eVariants from the conditional analysis (secondary signal, light grey) for each eGene. Negative/positive distances denote that the variant is located upstream/downstream of the TSS with regard to the direction of transcription. (**B**) Boxplot of the absolute distance of primary and secondary signals to the TSS. Significance was assessed by the Mann-Whitney-U-Test (P-value < 8.05 x 10^−43^).

We then characterized the 3,751 independent signals with respect to their significance and position regarding the regulated eGenes (**Fig 2A**). Signals were widely distributed around the transcription start sites (TSSs) of the respective eGenes with more significant eVariants clustering closer to the TSS (P-Value linear model 2.1 x 10^−17^). Interestingly, more than half (2,286/3,751) of the independent signals were located downstream of the respective TSSs. Of note, the most significant eVariants of the primary analysis were significantly located closer to the TSSs (median: 32,743 bp, SD: 182,220) when compared with eVariants identified by the conditional analysis (median: 105,418 bp, SD: 294,134; Mann-Whitney-U-Test P-value < 8.05 x 10^−43^) (**Fig 2B**).

To estimate variation in gene expression regulation in-between tissues, we compared our retinal eQTL results with the Genotype-Tissue Expression (GTEx) project version 8 summary statistics. These include gene expression and eQTL data regarding 49 tissues and cell types [10]. In comparison with the 16,766 autosomal genes included in our analysis, GTEx detected more expressed genes throughout all tissues (mean: 23,857.49, SD: 1,988.58). Further, the percentage of eGenes in GTEx varied widely from 5.11% (see “Kidney cortex”, n = 73) to 69.37% (see “Cells cultured fibroblasts”, n = 483) (**S2 Data**). The four GTEx tissues Pancreas (n = 305), Colon sigmoid (n = 318), Testis (n = 322), and Stomach (n = 324) have a comparable sample-size to our retinal database and showed a mean percentage of eGenes among all expressed genes of 44.23% (SD: 7.29). Interestingly, eGenes identified in retina were often eGenes in other tissues (mean 1,777.53, SD: 425.23).

### Regulatory cluster analysis in retinal tissue

In retinal tissue, 99,165 (24.6%) of the 403,151 unique eVariants regulate the expression of more than one eGene. This raises the question whether these variants are distributed arbitrarily over all autosomal chromosomes or rather cluster in distinct genomic regions. Furthermore, we tested whether the potential clusters originate from overlapping linkage disequilibrium (LD) structures in-between genetic signals or whether the same genetic signal regulates several genes, for example affecting a transcription factor binding site. To answer these questions, we applied a two-step protocol.

First, we filtered the eQTL dataset for eVariants regulating three or more eGenes. These variants were subsequently merged into potential regulatory clusters, by combining variants located on the same chromosome within a randomly chosen distance of less than 1 Mbp. Together, we identified 96 genomic regions, most of which (87/96) cluster in negative G-bands or in lightly stained bands (“gpos25” and “gpos50”) (**S3 Data**). The cluster sizes varied widely from 1 bp (1:46214495–46214495, 2:61162474–61162474, 3:150943708–150943708, 11:727310–727310, 12:120463130–120463130, 16:19584627–19584627 and 20:63253304–63253304), each containing a single eVariant regulating several genes, to 6,439,289 bp for cluster 6:26678284–33117573 including 51 regulated genes. The latter cluster comprised the most eVariants (n = 11,757).

**Fig 3:**
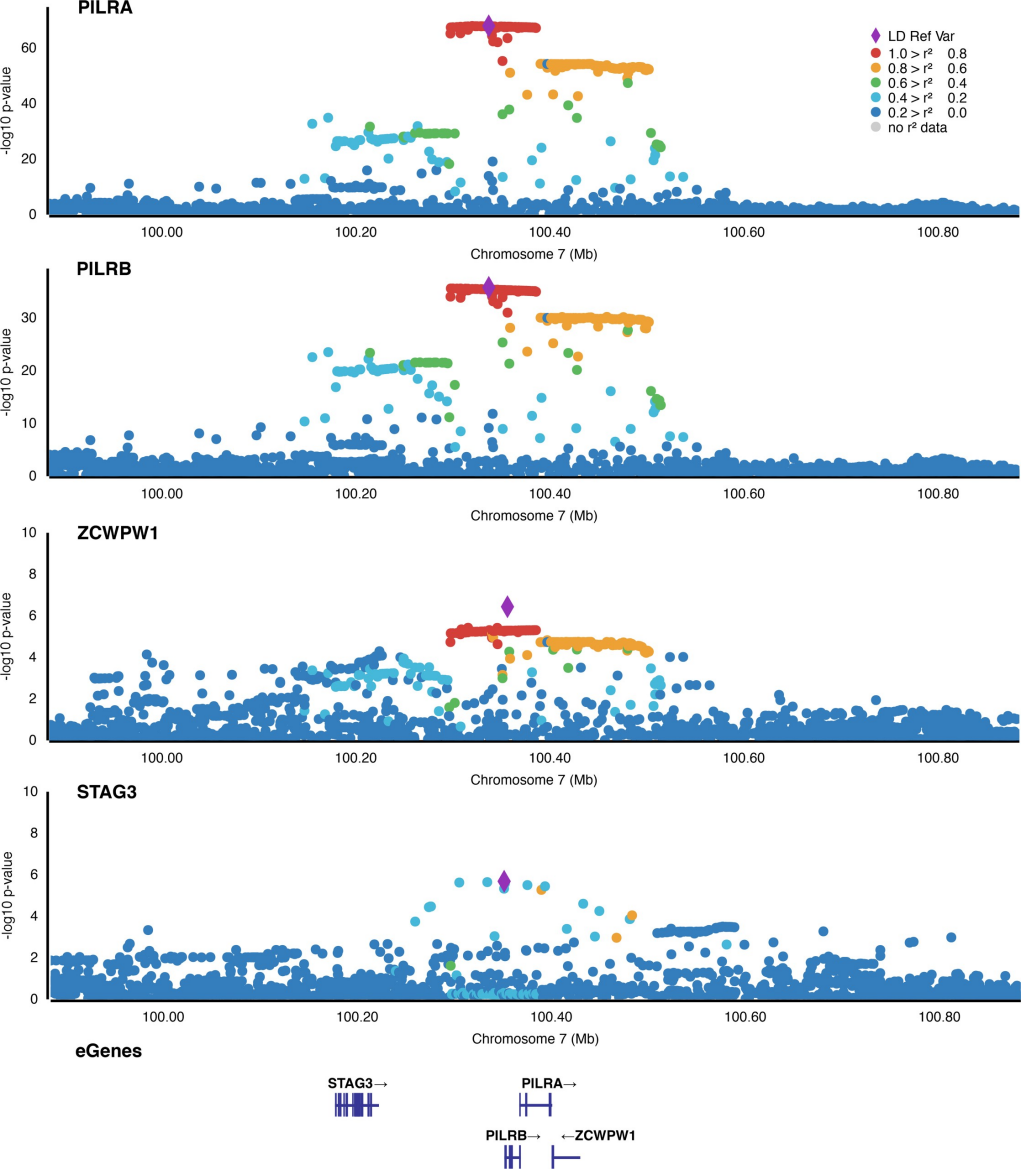
Locuszoom plots of the genetic signals regulating genes at the cluster 7:99418220–100808585. Shown are the nominal P-Values of all local variant—gene associations regarding the genes *PILRA*, *PILRB*, *ZCWPW1*, and *STAG3* in the cluster 7:99418220–100808585. Variants are colored according to their linkage disequilibrium (LD) with the respective most significant eVariant. LD data were retrieved selecting the EUR population and plots were generated using https://my.locuszoom.org/.

Second, we wanted to know whether these clusters represent functional units each regulated by the same genetic signal or whether they are generated by overlapping LD structures. To this end, we analyzed the co-localization of eQTL signals for each gene pair within a regulatory cluster using *coloc* [11]. A posterior probability threshold of at least 0.8 was applied to identify eGenes, which are regulated by the same genetic signal (**S4 Data**). Altogether, 2,938 possible gene pairs within the 96 regulatory clusters were analyzed for co-localization. Of these, 266 gene pairs showed posterior probabilities ≥ 0.8 and in 31 of the 96 clusters genetic signals regulated at least three genes (**S3 Data**). For example, the regulatory cluster 7:99418220–100808585 encompasses twelve eGenes but contains several genetic signals (**S2 Fig**). One of these regulates the three genes *PILRA*, *PILRB*, and *ZCWPW1*, which is demonstrated by co-localization probabilities ≥ 0.8 within all possible gene pairs. Other genes located within 7:99418220–100808585, for example *STAG3*, show co-localization posterior probabilities of < 0.03 with regard to *PILRA*, *PILRB*, and *ZCWPW1* and are therefore thought to be regulated by a different genetic signal (**Fig 3**). Interestingly, the largest cluster, namely 6:26678284–33117573, includes 51 different genes but only one genetic signal regulates more than 2 genes, pointing to a complex LD structure within this locus.

### eVariants associated with complex eye diseases and traits

In a final step, we explored the combined retina eQTL dataset by analyzing whether eVariants in retinal tissue are associated to any complex eye disease or trait. We integrated results from 16 published GWAS investigating 12 distinct ocular traits and disease entities (**S5 Data**). This collection comprises eye related GWAS at the time of our data search and was meant to cover as many potential effects as possible. The number of GWAS variants with genome-wide significance varied per phenotype from 3 (see “diabetic retinopathy”) to 251 (see “intraocular pressure”). Together, 665 of 716 variants were included in the data analysis (**S6 Data**). Of these, 25 variants were associated with more than one trait investigated. Sixty-five of the 665 unique GWAS variants appear to be regulating gene expression in retinal tissue (**S7 Data**). Remarkably, the disease or trait associated variants were significantly more often eVariants (9.77%, 65 of 665) in comparison to a set of all analyzed variants in this study (4.64%, 403,151 out of 8,686,883) (Fisher's exact test, P-Value: 4.78 x 10^−09^). It is noteworthy that GWAS variants (mean info score 0.972, SD: 0.054) were significantly better imputable than non GWAS variants (mean info score 0.957, SD: 0.057) (Mann-Whitney U-Test P-Value 3.7 x 10^−12^). Furthermore, eVariants (mean info score 0.974, SD: 0.054) were also better imputable in comparison to non-eVariants (mean info score 0.956, SD: 0.074) (Mann-Whitney U-Test P-Value < 2.2 x 10^−16^).

Moreover, we assigned RegulomeDB [12] ranks to all GWAS variants to test for overlaps with potential regulatory DNA elements like DNase hypersensitivity or transcription factor binding sites. The seven-level functional score is based on a synthesis of data derived from various sources: category 1 variants are very likely to affect binding and are linked to gene expression of a target gene, while category 7 variants lack evidence for any functional relevance. Notably, the percentage of GWAS variants assigned to the RegulomeDB ranks one to five is higher in each category in comparison to all variants included in our study (**S8 Data**). Nevertheless, 7 out of the 65 (10.6%) eVariants regulating gene expression in retina are associated with at least one ocular phenotype and were assigned the RegulomeDB rank 1 pointing to potential effects on gene regulation in other tissues as well.

**Fig 4:**
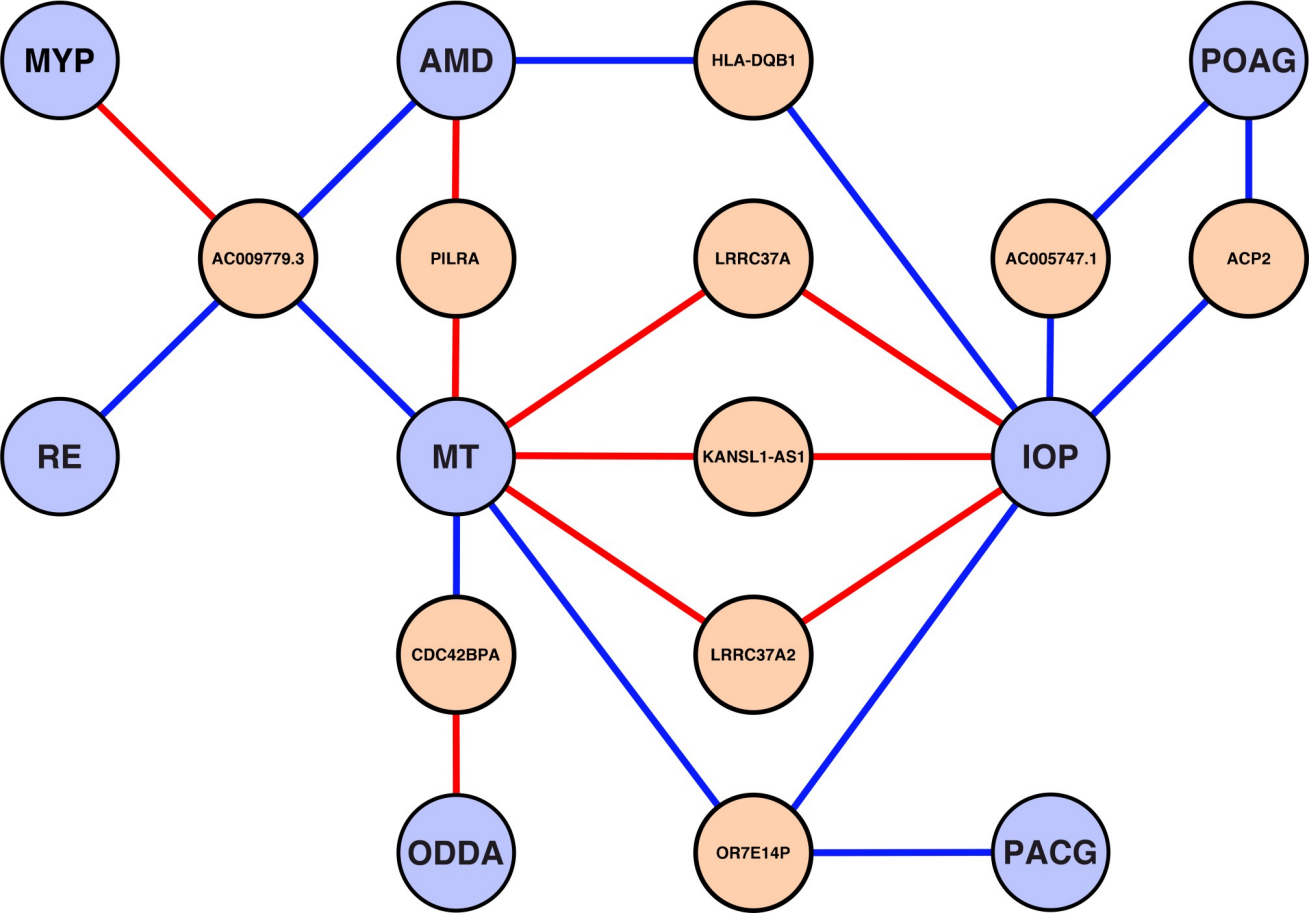
eGenes regulated by multiple complex eye disease- or trait-associated variants. Ten eGenes (orange) were regulated by genome-wide significant GWAS variants of at least two different complex eye diseases or traits (light blue). Connective lines are colored according to the eQTL effect direction of the risk-/trait- increasing allele. Red lines reflect higher gene expression whereas blue lines represent downregulation of expression. AMD = age-related macular degeneration; IOP = intraocular pressure; MT = macular thickness; MYP = myopia; ODDA = optic disc—disc area; PACG = primary angle closure glaucoma; POAG = primary open-angled glaucoma; RE = refractive error.

Overall, we identified 80 unique eGenes whose regulation is potentially disease- or trait-associated (**S7 Data**). Almost half of these (32 of 80 genes) are non-protein coding. Of note, 10 genes are regulated by eVariants associated with more than one disease or trait (**Fig 4**). For example, lower expression of non-annotated protein coding gene *AC009779*.*3* is potentially associated with increased risk for AMD, refractive error (RE), and increased macular thickness (MT) while elevated gene expression of *AC009779*.*3* is associated with an increased risk of myopia (MYP).

## Discussion

In this study, we combined genotypes and RNA sequencing data from 311 healthy retinal tissue to explore the regulatory architecture of gene expression in this highly differentiated and specialized tissue. The eQTL analysis pointed to 3,007 out of 16,766 autosomal retinal genes to be genetically regulated. Strikingly, gene expression regulation was most prominent in the categories of pseudogenes and lncRNAs. Mapping the location of the most significant independent signals relative to the organizational structure of the genes demonstrated a high density of the signals immediately adjacent to the respective transcription start sites although some were as far away as 10 kb and more. The latter finding should caution the interpretation of GWAS signals and should draw attention to the possibility that lead genetic variants may not necessarily exert effects on the gene physically closest but may instead act in a long range mode. Further, we identified regions in the genome harboring regulatory clusters of eGenes co-regulated by the same genetic signal. Finally, we have queried the retina eQTL database with GWAS results of a number of prevalent complex eye diseases and traits and have identified potential pleiotropic eGenes shared between the various disease etiologies. This latter aspect may prove critical for gaining a deeper insight into molecular mechanisms of a specific disease by considering pathways which involve functional properties of genes that contribute to various other diseases or ocular traits.

A comparison of our findings with those of the GTEx project [10] aimed to see if gene expression regulation in retinal tissue generally differs from other tissues. Overall, GTEx algorithms consider more genes to be expressed for each tissue leading to an increased relative percentage of eGenes among all expressed genes. This may be due to the comparison of a single streamlined project with a mega-analysis approach as done in our study combining raw data that originated from three different study sites. A study-by-study comparison showed that 2,412 genes were exclusively found to be expressed in only one or two of the three retinal datasets. This in line with a former study in which we performed an eQTL mega-analysis of four datasets investigating liver tissue. Here, we observed that only 53.5 to 80.2% of eQTL were shared in-between single datasets [13]. Although the same kind of tissue was investigated, a number of secondary effects such as handling of probes and methodical differences in data generation may greatly influence the outcome. An alignment of primary sequence data, as was done in this study, is highly recommended to harmonize independent datasets [14,15].

In our combined dataset over 31% of all expressed lncRNAs and pseudogenes in the retina are genetically regulated. This might be attributable to recent advances in genome research, which led to an increased discovery of non-coding DNA regions [16]. Alternatively, these findings in retina could be due to the co-localization with eQTL in clusters of retina-specific enhancers [17,18]. Specifically, lncRNAs and pseudogenes are still poorly characterized but are thought to have a multitude of regulatory functions through various mechanisms [19–22].

Our data suggest that a number of retinal eVariants regulate several genes and that this phenomenon is clustering in distinct regions in the human genome. The largest of these clusters is located on chromosome 6 (cluster ID: 6:26678284–33117573) and includes at least 51 eGenes. This cluster overlaps with the major histocompatibility complex (MHC), which plays a key role in immune response [23]. Interestingly, within this region the eVariant rs9271367 significantly regulates seven genes in retinal tissue (*TSBP1-AS1*, *HLA-DRB5*, *HLA-DRB6*, *HLA-DRB1*, *HLA-DQB1*, *HLA-DQA2*, and *HLA-DOB*). Co-localization analysis revealed that the genetic signals associated with these genes do not overlap (coloc probability < 0.8). This observation emphasizes that LD structures may play a role when investigating shared mechanisms in gene expression regulation. Our co-localization analysis further revealed that 31 of the beforehand detected regulatory cluster regions harbour genetic signals which regulate at least three genes. This is the case for genes *PILRA*, *PILRB* and *ZCWPW1* located on chromosome 7 (cluster ID: 7:99418220–100808585). Topological chromatin interactions were previously reported for this regulatory cluster [24]. Still, gene expression regulation can be tissue-specific and, so far, no chromosome conformation capture data are available for human retinal tissue [25,26]. It will be of interest to collect further data on topological chromatin interactions in relation to our results pointing to several genomic loci harbouring co-regulated genes. When it comes to targeted therapy, knowledge about shared regulatory mechanisms will help to appreciate side effects when addressing a specific gene, but will also suggest potential interaction partners with regard to the gene of interest.

To obtain insight into phenotype-associated processes, we investigated retinal gene expression regulation in the context of shared genetics between complex eye diseases and traits. To avoid a selection bias, we considered currently available GWAS studies investigating any type of ocular phenotypes. To this end, we identified 65 of 665 GWAS variants to regulate gene expression of at least one neighbouring gene. In three of the diseases and traits, namely AMD, diabetic retinopathy (DR), and MT, phenotypic expressions appear to intuitively reflect retinal lesions. We also included traits which may only indirectly affect the retina such as intraocular pressure (IOP). Interestingly, the retina-associated diseases and traits showed no enrichment of retinal eVariants (AMD: 4 of 41, DR: 0 of 3, and MT: 23 of 129) in comparison to the second group of diseases and traits with no direct link to retinal lesions (see also **S5 Data**). This suggests that apart from gene expression other mechanisms may explain GWAS association signals. Alternatively, functional expression of GWAS signals may occur outside the retina, which is in line with the high proportion of variants assigned to RegulomeDB rank 1. The latter argument is further supported by a recent study which demonstrated that AMD genetics influence gene expression throughout the whole body and that systemic processes might be relevant for disease aetiology [27]. So far, there are no eQTL data for tissues such as the choriocapillaris/choroid or the retinal pigment epithelium (RPE), two tissues intimately involved in retinal functionality. Tissue- or cell-specific effects may contribute to disease pathologies, but will ultimately require the respective eQTL data on a cellular resolution, which can be investigated by single cell RNA-Seq. Furthermore, one needs to consider that gene expression in a diseased tissue may well be different from gene expression in healthy tissue. We therefore want to point out that our current study can be hypothesis-generating with regard to disease aetiology, but may not be suited to draw direct conclusions for processes occurring beyond first disease onset.

The knowledge of retinal eGenes for ocular complex eye diseases and traits opens up the possibility to search specifically for pleiotropic effects. In turn, this may help to gain information on defined disease aetiologies. In our study, we show overlapping eGenes for eight traits, with most trait pairs sharing one to two eGenes. Strikingly, IOP and MT share four eGenes of which three (*KANSL1-AS1*, *LRRC37A2*, and *LRRC37A*) are located in a single regulatory cluster. The LRRC37 gene family seems to be evolutionary conserved, although their function is still unknown [28]. Another interesting finding is *AC009779*.*3* which is also only poorly characterized so far. The regulation of expression is potentially associated with four phenotypes, inlcuding AMD, MT, MYP, and RE. *AC009779*.*3* is a paralogue to *BLOC1S1*, which is known to belong to the BLOC-1 complex, a complex required for the biogenesis of lysosome-related organelles and the correct formation of melanosomes [29].

It is of note that many of the retinal eGenes, which are potentially associated with GWAS results, are non-coding (32 out of 80) or have not yet been assigned a HGNC symbol [30] (e.g. *AC009779*.*3* and *AC005747*.*1*). This could point to potential new mechanisms in disease processes but needs further experimental validation. Unfortunately, summary statistics are still unavailable for many GWAS datasets which impedes with a more detailed investigation of co-localized signals and thus an evaluation of possible pleiotropic effects. To this end, our analysis of GWAS signals in the context of retinal eQTL provides a starting point for further investigations.

Taken together, this study generated the largest retinal eQTL database to-date based exclusively on healthy tissue samples and provides insights into expression regulation of this unique tissue. It also offers a valuable resource for further work into functional aspects of associations of genetic variability in complex diseases of the retina. Specifically, we show that the eQTL data are useful to learn more about regulated genes associated with diseases and traits of the eye and thus offering gene candidates which may play a role in prevalent retinal disease aetiologies.

## Methods

### Ethics statement

In accordance with the tenets of the Declaration of Helsinki, postmortem human donor eyes were collected at the Ludwig-Maximilians-University Munich (termed the Regensburg dataset), the University Hospital Cologne (Cologne dataset), and the Minnesota Lions Eye Bank (Bethesda dataset) after informed consent from the donor or next of kin was obtained. Each study was approved by the corresponding local Institutional Review Board and application numbers were: MUC73416 (Regensburg dataset), 14–247 (Cologne dataset), and FWA00000312 (Bethesda dataset). All investigated samples in this study were approved for research use. In case of the Bethesda dataset, data were retrieved de-identified and no further IRB approval for the conducted analysis was required.

### Study subjects

The organ donors died of various reasons (**S9 Data**). Only clinically asymptomatic retinal samples with no sign of retinal pathology were included.

For the Regensburg dataset, globes were prepared for harvesting of corneoscleral buttons as described before [31]. After slit-lamp evaluation of the cornea, whole eyes were thoroughly cleansed in 0.9% NaCl solution, immersed in 5% polyvinylpyrrolidone-iodine, and rinsed with sodium chloride solution. Corneoscleral discs were then harvested, and stored in 50-mL tissue culture flasks within 4 hrs after enucleation. At the same time, the residual globe was prepared and vitreous was removed. The retina was harvested using two sterile forceps and the retinal tissue was dissected at the optic nerve head. Thereafter, the retinal tissue was transferred to a 1.5ml Eppendorf cup and stored at -80°C.

For the Cologne dataset, eyes were obtained within 6 hours after death and retinal dissection was performed immediately afterwards. The retinal tissues were flash frozen in liquid nitrogen and stored at -80°C until further processing.

For the Bethesda dataset, donor eyes were enucleated within 4 h of death and stored in a moist chamber at 4°C until retinal dissection was performed. Tissue sections were flash frozen in liquid nitrogen and stored at –80°C until further processing.

### Genotype data

The genotype data analysis was performed for each set of data individually before generating a merged genotype table (**Table 1**). Genotypes of the Bethesda dataset were processed as described earlier [7]. Data processing before imputation for the Regensburg and Cologne datasets were performed as follows: First, a Principal component analysis (PCA) was carried out in R (version 3.3.1) [32] using *snpgdsPCA* [33] including 30,000 genetic variants of each sample and the corresponding genotype information of the 1000 Genomes Project reference panel (Phase 3, release 20130502) [34]. The first two principal components (PCs) were plotted to determine the ethnicity (**S3 Fig**) Only samples clustering next to the European (EUR) reference individuals were included to take into account the known variation of haplotype structures between populations. Furthermore, related samples (kinship coefficient > 0.80; then, only a single random sample of a related pair was kept for analysis) and samples with contradictions in inferred and reported gender were excluded. QC on the variant level included (1) consideration of autosomal variants only, (2) removal of monomorphic variants, (3) removal of SNPs with Hardy-Weinberg equilibrium (HWE) P-Value < 1 x 10^−6^, and (4) a call rate < 95%. Thereafter, allele frequencies were compared with the respective frequencies as calculated by the 1000 Genomes Project EUR samples. Genetic variants with frequencies deviating by more than 13% were excluded from further analysis. Next, genotypes were phased using *SHAPEIT2 (*version 2.r904) [35] and thereafter imputed to the 1000 Genomes Project Phase 3 reference panel (October 2014) [36] by IMPUTE2 (version 2.3.2) [37]. For post imputation QC, the output files were converted into a VCF format containing confidently imputed variants (quality threshold > 0.3) in an estimated allele dosage format. Genomic positions were updated to hg38 using UCSC *liftover* [38]. A linear regression comparing allele frequencies across the single datasets showed high consistency (R2 > 0.97).

The independently imputed genotypes of each dataset were merged into a single file which was filtered for minor allele frequency (MAF > 1%). This resulted in 8,686,883 genetic variants. Additionally, another genotype PCA was performed to include the first five PCs into the eQTL analysis.

### Gene expression data

The RNA-Seq data of each dataset were generated using library preparation protocols as given in **Table 1**. The evaluation of raw reads was performed for each dataset individually by applying a uniform protocol. Briefly, during all steps of the analysis *FastQC* (version 0.11.5) [39] and *MultiQC* (version 1.7.dev0) [40] were used to ensure the correctness of the conducted data processing steps. First, raw reads were trimmed for adapter sequences and low quality (SLIDING WINDOW 4:5; LEADING 5; TRAILING 5; MINLEN 25) using *Trimmomatic* (version 0.39) with the supplied Illumina TruSeq3 sequences [41]. Thereafter, the star aligner (version 2.7.1a) [42] was employed with ENCODE standard options to align the trimmed reads to an Ensembl version 97 (GRCh38.p13, including GENCODE version 31) [43] based reference genome. The *RSEM* toolbox (version 1.3.1) [44] calculated estimated gene expression counts using standard options. The Regensburg dataset required two additional parameters about the fragment length distribution as it was based on single-end reads (fragment-length-mean 155.9 and fragment-length-sd 56.2). These values were obtained by calculating the mean fragment length distribution of 30 samples taken randomly from the Cologne and Bethesda datasets. The estimated counts were then normalized to the trimmed mean of M-Values (TMM) using the *tmmnorm* function of the *edgeR* package (version 3.16.5) [45] in R. Expression values were converted to counts per million (CPM) and expressed genes were retained (CPM > 1 in 10% of the samples). A PCA was performed with the help of the *prcomp* function in R to identify and to remove potential outlier samples within the dataset.

After log2 transformation with an offset of 1, the expression files of the three datasets were merged into a single matrix containing only genes, which were expressed in all datasets. Afterwards, quantile normalization and ComBat [46], an empirical batch correction method, were employed to normalize the data in regard to the originating dataset (**S4 Fig**).

### eQTL calculations

Local eQTL were calculated based on linear regression models using *FastQTL* (version v2.184_gtex) [47] as implemented in the GTEx version 8 analysis pipeline [10]. The mapping window to detect local eQTL was defined as 1 Mbp up- and down-stream of the gene start and end sites as defined by Ensembl [43]. Age, gender, study site, post-mortem interval (PMI) and the first five PCs of the genotype PCA were included in the models as covariates. First, *FastQTL* was applied in the—*permute 1000 10000* mode to calculate beta distribution-extrapolated empirical P-values for each potential eGene. These P-values were then used to calculate gene-level Q-Values using the false discovery rate (FDR) approach from Storey et al. (2003) as implemented in the R package *qvalue* (version 2.6.0) [48]. The lambda parameter was set to 0.85. A Q-Value threshold of < 0.05 was applied to identify eGenes with at least one significant eVariant. To detect all significant eVariants, a genome-wide empirical P-value threshold was defined as described in [10]. This threshold was used to calculate a nominal P-value threshold for each gene based on the beta distribution parameters from *FastQTL*. Thereafter, *FastQTL* was run in normal mode to calculate nominal P-values of all local gene—variant associations. Linear regression model nominal P-values below the beforehand defined gene specific threshold were considered significant. Furthermore, we used *FastQTL* to identify independent secondary signals by adjustment for the most significant corresponding eVariant. Thereafter, the eQTL were re-calculated and remaining significant eVariants were considered to represent a further independent signal. The most significant independent eVariant was then added to the covariate file. This approach was repeated until no additional independent signals were found. The gene specific nominal P-value threshold was applied during the procedure.

### Comparison of eQTL in GTEx v8 and retinal tissue

The GTEx summary statistics containing all genes analyzed (.egenes.txt.gz files) were downloaded from https://www.gtexportal.org/home/datasets (accessed April 24^th^ 2020). These files were processed per tissue and first filtered to contain exclusively genes located on autosomes. For the remaining genes, an adapted Q-Value was calculated as described above and a Q-Value 0.05 was used to determine significant eGenes.

### Regulatory cluster analysis

Regulatory clusters were defined as genomic regions containing multiple genes regulated by the same eQTL signal. This analysis required a two-step protocol. First, all significant eVariants were filtered for variants regulating three or more eGenes. These variants were subsequently merged into potentially regulatory clusters by combining variants located on the same chromosome within a chosen distance of less than 1 Mbp. In a second step, all genes within a cluster were investigated for co-localization of their associated variants. This was performed using the *coloc*.*abf* function of the *coloc* package [11] in R with standard options. The method utilized the respective eQTL nominal P-values and effect sizes as well as the standard deviation of the expression for the investigated genes. In addition, the MAF was supplied for all variants. Next, the posterior probability that the same eQTL signal is regulating both genes (H4) was extracted from the co-localization analysis. If the analyzed gene pair did not share any overlapping variants within the 1 Mbp window, the H4 value was assigned to 0. Then a heatmap for each potential cluster was generated including the posterior probabilities of all possible gene pairs. This heatmap was reordered using the R function *hclust* with standard options to highlight co-localization of multiple genes with each other. eGenes, whose associated variants showed a co-localization probability of at least 0.8 were defined as having the same underlying eQTL signal. Finally, eGenes were grouped based on their probability to be regulated by the same genetic signal.

### GWAS of complex eye diseases and traits

To identify relevant GWAS, we searched PubMed (https://www.ncbi.nlm.nih.gov/pubmed/) and the NCBI GWAS catalog [1] (accessed May 2019) for common eye diseases and traits by keywords such as “macular”, “refraction”, and “astigmatism”. The subjects included within each study had to be predominantly of European descent. Smaller studies were excluded if a more recent GWAS with extended sample-sizes was available. We only included genome-wide significant variants (P-Value < 5 x 10^−08^) in the analysis. Additionally, QC of variants encompassed a comparison of allele frequencies to the 1000 genomes reference panel and a linkage analysis. Strongly linked variants (R^2^ > 0.5) were removed and only the most significant variant was kept.

## Supporting information

S1 Fig Exemplary eQTL plots. Shown are violin plots of the three most significant eQTL in retinal tissue: (**A**) rs145885457—*TSPAN11*, (**B**) rs384490—*AC108448*.*3*, and (**C**) rs5751775—*AP000350*.*6*. (**D**) The eQTL rs141866579—*AL590399*.*1* showed the highest nominal P-Value (2.51 x 10^−03^) for regulation of a significant eGene in the analysis. The sample size (n) for each genotype is given below the respective alleles.
(TIF)

S2 Fig Heatmap including co-localization posterior probabilities for all genes located in the cluster 7:99418220–100808585. All variant—gene associations of the 12 eGenes located in the cluster 7:99418220–100808585 were analyzed for co-localization using coloc [11]. Shown are the posterior probabilities that the eQTL signal for one gene is overlapping with the respective signal regulating the other gene. Posterior probabilities above 0.8 were colored in red with increasing intensity.
(TIF)

S3 Fig Genotype Principal Component Analysis. 30,000 autosomal variants were randomly selected and the genotypes of those variants were extracted from the three datasets (Regensburg, Cologne, and Bethesda). In addition, we extracted the genotypes from samples of European (EUR), African (AFR), South Asian (SAS), and East Asian (EAS) ancestry from the 1000 Genomes Project and performed a PCA. Plotted are the first three PCs of all 311 included retinal tissue samples and the results of the populations EUR, AFR, and SAS for (**A**) PC1 and PC2, (**B**) PC1 and PC3, and (**C**) PC2 and PC3.
(TIF)

S4 Fig Normalization process for gene expression data. A PCA was conducted on the merged gene expression data from the three datasets (Regensburg, Cologne, and Bethesda), at three different consecutive normalization steps including (**A**) log2 transformed CPM values, (**B**) quantile normalized data, and (**C**) after adjustment for the known batch effect originating from the different study sites using ComBat [46].
(TIF)

S1 Data Independent eQTL signals (Q-Value < 0.05). (XLSX)

S2 Data Comparison of autosomal gene expression and eGene count in retina and GTEx tissues (Q-value < 0.05). (XLSX)

S3 Data Overview of regulatory clusters in retinal tissue. (XLSX)

S4 Data Posterior probability for co-localization of genetic signals underlying two eGenes. (XLSX)

S5 Data Complex eye diseases and traits investigated in this study. (XLSX)

S6 Data Genome-wide significant GWAS variants of complex eye diseases and traits. (XLSX)

S7 Data eVariants associated with complex eye diseases and traits (Q-Value < 0.05). (XLSX)

S8 Data Distribution of RegulomeDB ranks in regard to the eQTL results in retinal tissue. (XLSX)

S9 Data Tissue donor and sample attributes. (XLSX)
