# Supplementary figures and images for: A mega-analysis of expression quantitative trait loci in retinal tissue
Source: PLoS Genet. 2020 Sep 1;16(9):e1008934. doi: 10.1371/journal.pgen.1008934 (PMC7462281; doi:10.1371/journal.pgen.1008934)

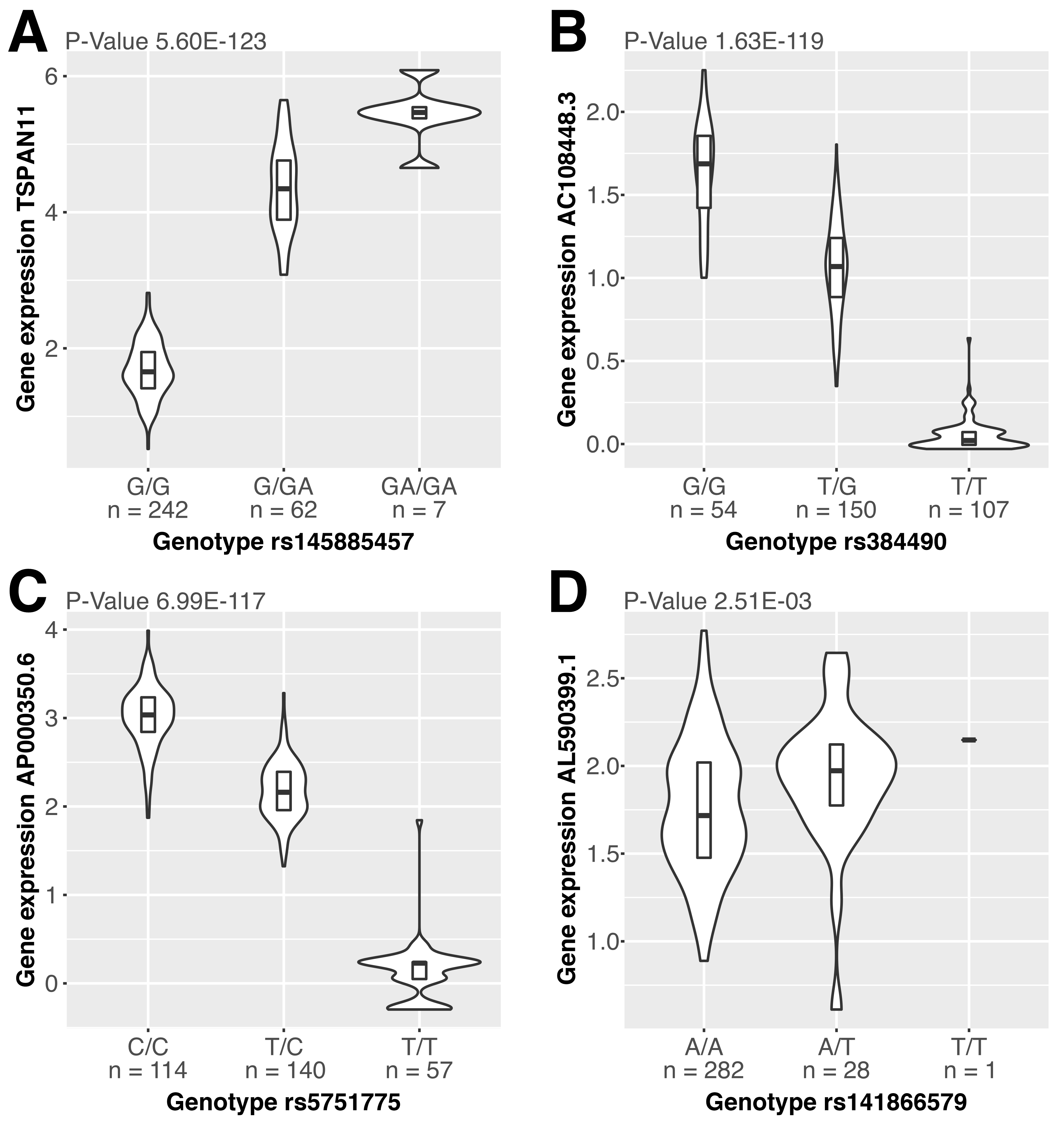

Supplement: S1 Fig — Shown are violin plots of the three most significant eQTL in retinal tissue: (A) rs145885457—TSPAN11, (B) rs384490—AC108448.3, and (C) rs5751775—AP000350.6. (D) The eQTL rs141866579—AL590399.1 showed the highest nominal P-Value (2.51 x 10^−03^) for regulation of a significant eGene in the analysis. The sample size (n) for each genotype is given below the respective alleles. (TIF) [file pgen.1008934.s001.tif]

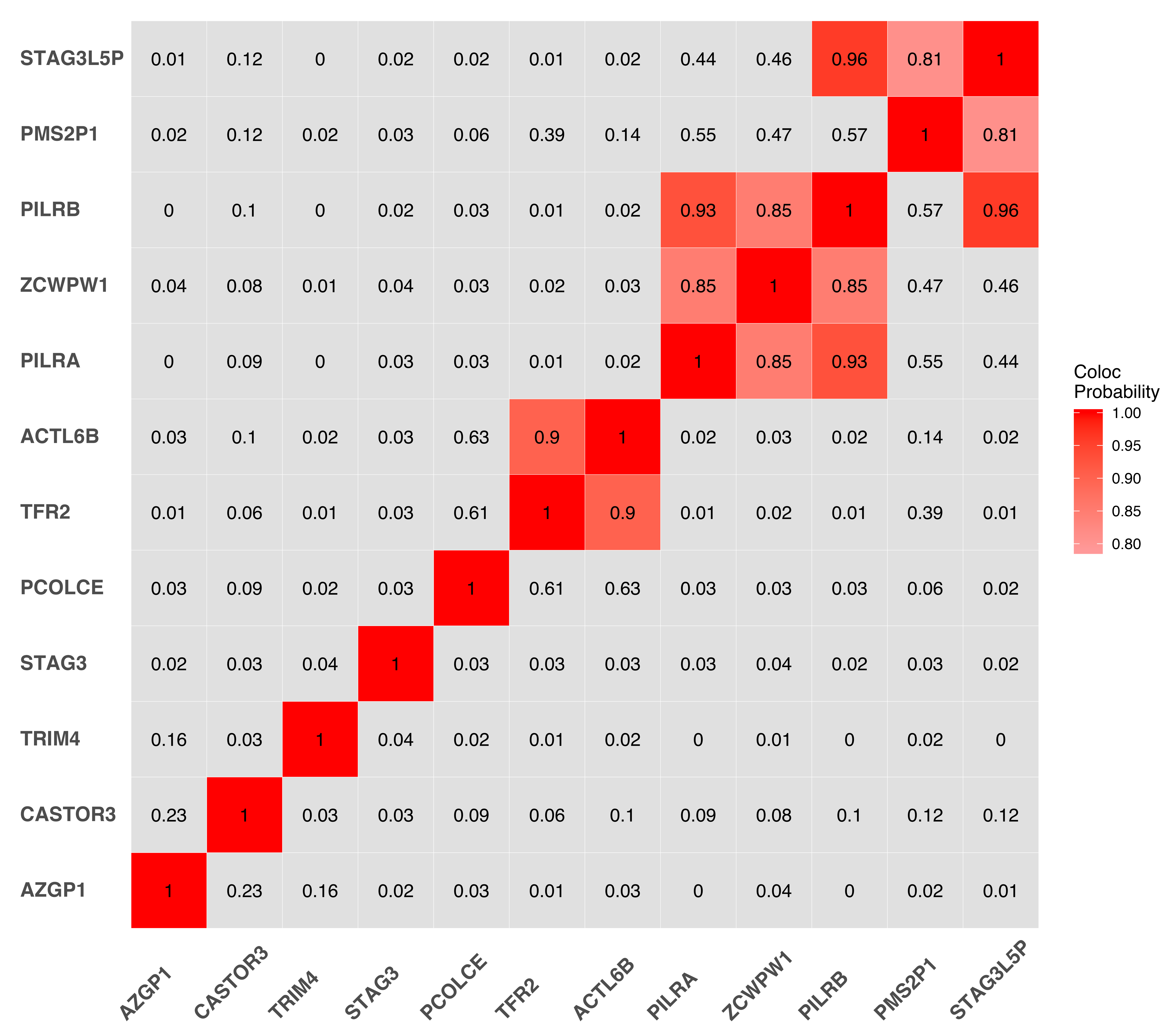

Supplement: S2 Fig — All variant—gene associations of the 12 eGenes located in the cluster 7:99418220–100808585 were analyzed for co-localization using coloc [11]. Shown are the posterior probabilities that the eQTL signal for one gene is overlapping with the respective signal regulating the other gene. Posterior probabilities above 0.8 were colored in red with increasing intensity. (TIF) [file pgen.1008934.s002.tif]

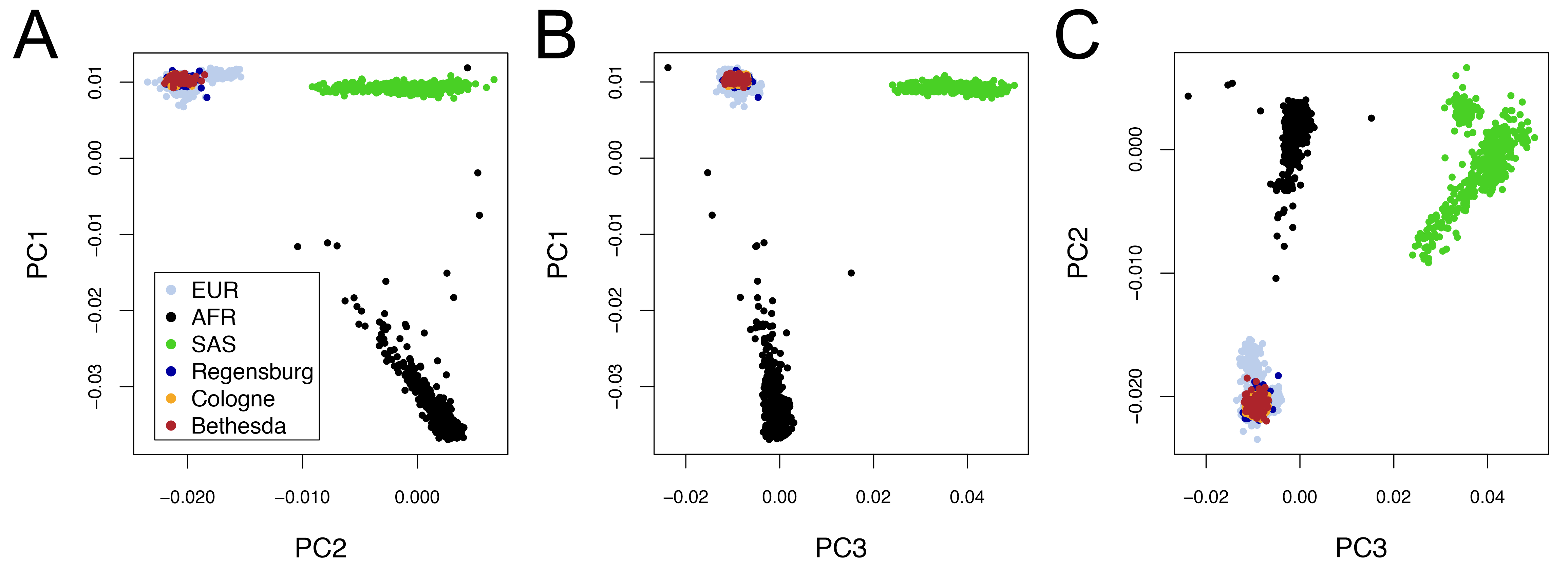

Supplement: S3 Fig — 30,000 autosomal variants were randomly selected and the genotypes of those variants were extracted from the three datasets (Regensburg, Cologne, and Bethesda). In addition, we extracted the genotypes from samples of European (EUR), African (AFR), South Asian (SAS), and East Asian (EAS) ancestry from the 1000 Genomes Project and performed a PCA. Plotted are the first three PCs of all 311 included retinal tissue samples and the results of the populations EUR, AFR, and SAS for (A) PC1 and PC2, (B) PC1 and PC3, and (C) PC2 and PC3. (TIF) [file pgen.1008934.s003.tif]

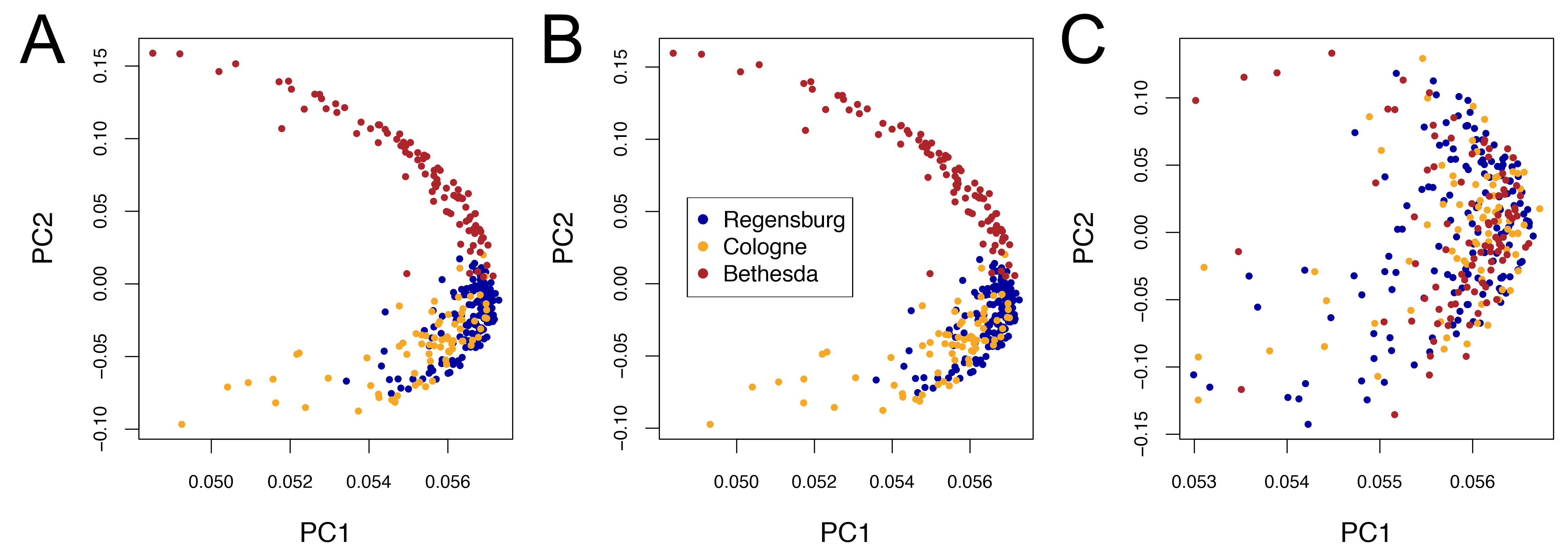

Supplement: S4 Fig — A PCA was conducted on the merged gene expression data from the three datasets (Regensburg, Cologne, and Bethesda), at three different consecutive normalization steps including (A) log2 transformed CPM values, (B) quantile normalized data, and (C) after adjustment for the known batch effect originating from the different study sites using ComBat [46]. (TIF) [file pgen.1008934.s004.tif]
